# Associations between physical multimorbidity patterns and common mental health disorders in middle-aged adults: A prospective analysis using data from the UK Biobank

**DOI:** 10.1016/j.lanepe.2021.100149

**Published:** 2021-06-22

**Authors:** Amy Ronaldson, Jorge Arias de la Torre, Matthew Prina, David Armstrong, Jayati Das-Munshi, Stephani Hatch, Rob Stewart, Matthew Hotopf, Alexandru Dregan

**Affiliations:** a Institute of Psychiatry, Psychology and Neuroscience (IoPPN), King's College London, London, UK; b CIBER Epidemiology and Public Health (CIBERESP), Madrid, Spain; c Institute of Biomedicine (IBIOMED), University of Leon, Leon, Spain; d Health Service and Population Research Department, Institute of Psychiatry, Psychology and Neuroscience, King's College London, London, UK; e Department of Primary Care and Public Health Sciences, King's College London, London, UK; f ESRC Centre for Society and Mental Health, King's College London, London, UK; g South London and Maudsley NHS Foundation Trust, London, United Kingdom

**Keywords:** Multimorbidity, Depression, Anxiety, Exploratory factor analysis

## Abstract

**Background:**

We aimed to identify specific patterns of physical multimorbidity, defined as the presence of two or more physical long-term conditions, and to examine the extent to which these specific patterns could predict future incident and persistent common mental health disorders (CMDs) in middle-aged adults enrolled in the UK Biobank.

**Methods:**

We assessed prospective associations between physical multimorbidity status at the baseline assessment (2006–2010) and depression and anxiety ‘caseness’ according to the Patient Health Questionnaire (PHQ)-9 and the Generalised Anxiety Disorder Assessment (GAD)-7 at the follow-up assessment (2016) in 154,367 middle-aged adults enrolled in the UK Biobank (median age: 57 years, interquartile range *=* 50–62 years, 56.5% female, mean duration of follow-up: 7.6 years, standard deviation = 0.87). Patterns of physical multimorbidity were identified using exploratory factor analysis. Logistic regression was used to assess prospective associations between physical multimorbidity patterns at baseline and both incident and persistent depression and anxiety at follow-up.

**Findings:**

Compared to those with no physical multimorbidity, having two (adjusted odds ratio (aOR) =1.41, 95%CI 1.32 to 1.53), three (aOR = 1.94, 95%CI 1.76 to 2.14), four (aOR = 2.38, 95%CI 2.07 to 2.74), and five or more (aOR = 2.89, 95%CI 2.42 to 3.45) physical conditions was prospectively associated with incident depression at follow-up in a dose response manner. Similar trends emerged for incident anxiety, persistent depression, and persistent anxiety, but associations were strongest for incident CMDs. Regarding specific patterns of physical MM, the respiratory pattern (aOR = 3.23, 95%CI 2.44 to 4.27) and the pain/gastrointestinal pattern (aOR = 2.19, 95%CI 1.92 to 2.50) emerged as the strongest predictors of incident depression. Similar results emerged for incident anxiety.

**Interpretation:**

These findings highlight patterns of physical multimorbidity with the poorest prognosis for both emerging and persisting depression and anxiety. These findings might have significant implications for the implementation of integrated mental and physical healthcare and facilitate the development of targeted preventative interventions and treatment for those with physical multimorbidity.

**Funding:**

AR is supported by Guy's Charity grant number EIC180702; JAT is funded by Medical Research Council (MRC) number MR/SO28188/1; AD is funded by Guy's Charity grant number EIC180702 and MRC grant number MR/SO28188/1. JD is part supported by the ESRC Centre for Society and Mental Health at King's College London (ES/S012567/1), grants from the ESRC (ES/S002715/1), by the Health Foundation working together with the Academy of Medical Sciences, for a Clinician Scientist Fellowship, and by the National Institute for Health Research (NIHR) Biomedical Research Centre at South London and Maudsley NHS Foundation Trust and King's College London and the National Institute for Health Research (NIHR) Applied Research Collaboration South London (NIHR ARC South London) at King's College Hospital NHS Foundation Trust. The views expressed are those of the author[s] and not necessarily those of the ESRC, NIHR, the Department of Health and Social Care or King's College London.

**Research in context**

**Evidence before this study**

There is a significant body of evidence to suggest that physical multimorbidity can lead to the development, or the persistence, of depression. However, less is known about how specific patterns of physical multimorbidity might impact future mental health, particularly in middle-aged adults. Previous studies that have investigated the impact of specific disease clusters on depression have clustered physical conditions *a priori* based on broad disease categories. Only one study to date has used exploratory methods to determine specific physical multimorbidity patterns in order to examine their impact on future depression in older adults in China. Moreover, no one has yet assessed the effects of physical multimorbidity patterns on anxiety specifically. In the current study, we used large-scale data from the UK Biobank to identify specific patterns of physical multimorbidity and to examine the extent to which these patterns prospectively associated with depression and anxiety in middle-aged adults in the United Kingdom.

**Added value of this study**

To our knowledge, this large-scale study was the first to identify patterns of physical multimorbidity and examine the extent to which these patterns predicted both new onset and persistent common mental health disorders in middle-aged UK adults. We reported a dose-response relationship between baseline physical multimorbidity status and incident depression and anxiety at follow-up. Similar results were found for persisting symptoms of common mental health disorders. We found that the respiratory and the pain/gastrointestinal patterns of physical multimorbidity were most strongly associated with incident depression and anxiety at follow-up.

**Implications of all available evidence**

The evidence taken together suggests that physical multimorbidity increases the risk of both new onset and persisting common mental health disorders. Moreover, there is increasing evidence that respiratory multimorbidity and multimorbidity where painful and gastrointestinal conditions cluster together might increase risk of future depression and anxiety considerably more than other physical multimorbidity disease clusters. These findings have implications for the implementation of integrated physical and mental healthcare services and highlight patient groups that might require targeted interventions.

Alt-text: Unlabelled box

## 1 Introduction

In the United Kingdom approximately 27% of adults in primary care have multimorbid conditions [1] (two or more co-existing health disorders) and this is set to rise considerably in the coming years [2]. Multimorbidity provides a considerable challenge to patients and their caregivers, clinicians, and the health and care systems given that multimorbidity is associated with physical functional decline [3], poor health-related quality of life [4], poor future health status [5] increased health care utilisation [6], and mortality [7]. However, the relationship between multimorbidity and mental health is more complex.

The relationship between physical multimorbidity and depression is generally thought to be bidirectional [8]. Most of the current evidence is, however, based on clinical and/or older populations where common mental health disorders are less well captured [9], [10], [11], [12], [13], [14]. Moreover, less is known about the association of specific patterns of physical multimorbidity with both emergent and persistent common mental health disorders (e.g., depression, anxiety) during mid-adult years, the period that sets the stage for multimorbidity patterns in older age. Research focusing on single conditions has found high rates of depression in patients with cancer, cardiovascular disease (CVD), chronic obstructive pulmonary disorder (COPD), stroke, diabetes, epilepsy, and Parkinson's disease [15,16], but reported rates of depression seem to vary considerably between medical illnesses [15]. This would imply that rates of incident and persistent depression might also vary between specific physical multimorbidity clusters.

Previous studies that have investigated associations between specific clusters of disease and depression have clustered physical conditions *a priori* based on broad disease categories [14,17,18]. To our knowledge only one study has adopted an exploratory approach to determine physical multimorbidity clusters, and the prospective associations between these clusters and depression in older adults in China [19]. Moreover, to date, no study has assessed the effect that physical multimorbidity might have on incident anxiety specifically.

In the current study, we aimed to assess associations between physical multimorbidity (disease count) and both emergent and persistent common mental health disorders (e.g., depression and anxiety). We then aimed to identify specific patterns of physical multimorbidity, and to examine the extent to which these patterns were prospectively associated with depression and anxiety among middle-aged adults using data from the UK Biobank.

## 2 Methods

### 2.1 Study design and participants

The UK Biobank is a large population-based prospective study established for the investigation of the determinants of disease in middle- and older-aged adults. Data were collected from more than 500,000 participants aged between 40 and 69 years from 22 different assessment centres across England, Scotland, and Wales between 2006 and 2010 [20]. Participants had to be registered with a general practitioner (GP) and live within 25 miles of an assessment centre to take part. Detailed accounts of sociodemographic, lifestyle, and medical information were gathered from all patients recruited to the study using a touchscreen questionnaire during the baseline assessment. Patients also provided information about medical diagnoses in a computer-assisted personal interview administered by trained interviewers. In 2016, an online mental health questionnaire was completed by 157,366 participants [21]. This questionnaire was completed online using a web-questionnaire platform and collected information relating to depression, generalised anxiety disorder, alcohol and substance misuse, post-traumatic stress disorder, mania, psychosis, and self-harm using both established psychometrics and self-report. All participants gave informed consent. The UK Biobank has ethical approval from the NHS National Research Ethics Service (16/NW/0274).

In the current study, the sample were selected based on those who completed the depression measure in the follow-up online mental health questionnaire. We stratified the analysis by depression and anxiety status at baseline in order to investigate (a) incident depression/anxiety in people with no depression/anxiety at baseline, and (b) persistent depression/anxiety symptomatology in those with depression/anxiety at baseline.

### 2.2 Baseline depression and anxiety

This study used multiple sources to determine depression at baseline: self-report, the Patient Health Questionnaire (PHQ)-2 (depression items) [22], and linked Hospital Episode Statistics (HES). A detailed description of how baseline depression was measured in the current study is provided in previous work assessing depression using UK Biobank data [23]. Anxiety at baseline was also measured using a combination of self-report, the PHQ-2 (anxiety items), and linked HES.

### 2.3 Exposure: physical multimorbidity

Physical long-term conditions were measured at the baseline assessment using both self-report lifetime diagnoses and linked hospital admission records (HES). Based on previous work on multimorbidity in large population samples, a total of 43 physical conditions were classified [24,25]. Self-report lifetime diagnoses were recorded during a nurse-led interview. Linked HES data were used to identify primary and secondary diagnoses of these conditions made before the UK Biobank baseline assessment. In the current study, after excluding psychiatric conditions and potentially non-chronic conditions (i.e., constipation), a total of 36 long-term conditions (e.g., asthma, cancer, epilepsy, diabetes) were used to assess physical multimorbidity at baseline. A comprehensive list of included conditions, the UK Biobank coding, and the *International Statistical Classification of Diseases and Related Health Problems, Tenth Revision (ICD-10)* codes for each condition are provided in Supplementary Material; Table S1. In the current study, an overall physical multimorbidity measure was defined using a cut-off of two or more conditions for each study participant [26]. To assess potential dose-response associations with mental health outcomes, we created an ordinal physical multimorbidity status variable grouping participants into; 0 or 1 condition (no multimorbidity); 2 conditions; 3 conditions; 4 conditions; 5 or more conditions.

### 2.4 Outcome: depression

The PHQ-9 was used to assess depression at follow-up [27]. The PHQ-9 is a nine-item questionnaire which scores each of the nine DSM-IV criteria for depression as ‘0’ (‘not at all’) to ‘3’ (‘nearly every day’). Scores range from 0-27. A score of 10 or higher is indicative of depression [28] and this was used to create a binary depression outcome variable. The PHQ-9 has been shown to be a valid and reliable diagnostic tool for possible depression in a variety of populations [29,30].

### 2.5 Outcome: anxiety

The Generalised Anxiety Disorder (GAD)-7 was used to assess anxiety at follow-up[31]. The GAD-7 is a seven-item questionnaire which asks participants to indicate the extent to which they had experienced certain anxiety symptoms (e.g., *‘feeling nervous, anxious, or on edge’, ‘worrying too much about different things’*) over the past two weeks on a four-point scale ranging from ‘0’ (‘not at all’) to ‘3’ (‘nearly every day’). Scores range from 0-21 with a score of 10 or higher indicating the presence of moderate to severe anxiety [31]. In the current study, this cut-off was used to create a binary anxiety variable. The GAD-7 has been shown to have good sensitivity and specificity for the diagnosis of common anxiety disorders in primary care [32].

### 2.6 Covariates

Several covariates were included, and their selection was informed by previous literature and theory. Sociodemographic variables such as age, gender, ethnicity (White/ethnic minority group), social deprivation, education level, and employment status are known to be associated with both physical multimorbidity and common mental health disorders and were therefore included as covariates [8,33,34]. Deprivation was based on Townsend deprivation indices[35] derived from aggregated data on car ownership, household overcrowding, owner occupation and unemployment. Higher scores were indicative of higher deprivation. Education level was coded into three categories: high (college or university degree), intermediate (A/AS levels or equivalent, O levels/GCSEs or equivalent), and low (none of the aforementioned). Employment status was also coded into three categories: employed, retired, unemployed/volunteer/carer. Health behaviours such as alcohol intake (number of units of alcohol consumed per week), smoking (current or past smoker), and physical activity, and factors related to health behaviour such as body mass index (BMI) are known to be associated with physical and mental health status so were included as covariates [8,36]. Physical activity was assessed with a modified version of the International Physical Activity Questionnaire that recorded total physical activity (e.g., mild, moderate, vigorous) performed over the previous seven days. Accordingly, participants were classified into four mutually exclusive categories: none, low (<600 metabolic equivalent (MET) minutes/week), moderate (600 to <3000 MET), or vigorous (3000+ MET). Height and weight were collected during the baseline assessment and used to derive BMI using the standard formula (kg/m^2^). The distance of time between the baseline assessment and the mental health follow-up questionnaire in years was also included as a covariate as it is plausible that differences in the duration between measurement of physical and mental health in the current study could affect the strength of the reported associations. Baseline depression was included as a covariate where anxiety was the outcome, and baseline anxiety was included as a covariate where depression was the outcome seeing as symptoms of these two conditions overlap considerably [37]. Baseline antidepressant use, which was measured using self-report (yes/no), was included as a covariate since these drugs may affect the likelihood of developing new onset depression and can also be prescribed for related conditions (e.g., pain, sleep disorders, fibromyalgia). Baseline levels of C-reactive protein (CRP) were included in the current study as a measure of chronic inflammation, known to be associated with both physical health and mental health [38]. Circulating levels of CRP were measured using high-sensitivity assays at the baseline assessment.

### 2.7 Statistical analyses

Variables were summarised as means and standard deviations, medians and interquartile ranges, and frequencies. Unadjusted, age- and sex-adjusted, and fully adjusted potential prospective associations between physical multimorbidity status and depression and anxiety outcomes were examined separately in participants with and without depression and anxiety respectively at baseline using multivariable logistic regression models. In fully adjusted models, we controlled for *a priori* confounders including age, sex, ethnicity, social deprivation, education level, employment status, BMI, smoking status, weekly alcohol intake, physical activity, CRP levels, the duration of time between baseline assessment and follow-up, use of antidepressants at baseline, baseline anxiety (depression as outcome), and baseline depression (anxiety as outcome).

We performed exploratory factor analysis (EFA) to identify associations between physical long-term conditions that exhibited physical multimorbidity patterns. This method of analysis has been used previously to identify associations among conditions under the assumption that they have commonly underlying aetiology and allows for a disease to belong to more than one multimorbidity pattern [19]. We applied the principal factor method based on a tetrachoric correlation matrix since physical conditions were coded as dichotomous variables in the current study. The suitability of the data to perform an EFA was assessed using the Kaiser-Meyer-Olkin (KMO) measure of sampling adequacy and Bartlett's Test of Sphericity. The number of factors identified was based on the shape of the screeplot, having an Eigenvalue >1, as well as parallel analysis. We used an oblique rotation of factor loading matrices, with each resulting factor loading representing the strength of association between the condition and the latent factor (i.e., multimorbidity pattern). A condition was considered to belong to a specific multimorbidity pattern if the rotated factor loading was at least +/-0.4^19^.

We assigned participants with multimorbidity to the specific multimorbidity patterns identified if they had reported a diagnosis of at least two of the diseases included in the pattern. All other patients were either assigned to a no multimorbidity (0 or 1 long-term condition) or an undefined multimorbidity (2 or more diseases not combined in the EFA) group. Logistic regression was used to assess unadjusted, age- and sex-adjusted, and fully adjusted prospective associations between physical multimorbidity patterns at baseline and depression and anxiety at follow-up in those with and without depression and anxiety at baseline respectively. We included multimorbidity patterns in the logistic regression models only if they included at least 1% of the population who had multimorbidity.

Data were missing for several variables: ethnicity (0.3%), deprivation (0.1%), education levels (0.6%), employment status (0.6%), BMI (0.2%), alcohol intake (25.5%), CRP levels (5.6%), and anxiety at follow-up (1.0%). As multivariable normality could not be assumed, multiple imputation using chained equations with 10 imputations was performed to deal with missing data [39]. Multiple imputation included outcome and exposure variables as well as all covariates in order to account for the complex interrelationships between all study variables. All fully adjusted analyses were based on imputed data.

All analyses were conducted in STATA 15.1 (Stata Corp LLP, College Station, TX).

### 2.8 Sensitivity analyses

Planned sensitivity analyses were also carried out to validate the study's primary analyses. We examined differences in exposure variables, outcome variables, and covariates between those who did and did not complete the follow-up online mental health questionnaire using independent t-tests and chi square tests. This analysis aimed to highlight potential differences between the analytical and the excluded sample which might introduce bias in the study findings.

Additional sensitivity analyses aimed to assess concurrent associations between physical multimorbidity and common mental health disorders, using different conceptualisations of physical multimorbidity. These analyses aimed to identify whether the association between physical multimorbidity and study outcomes varied according to the definition of the former. Firstly, we assessed cross-sectional associations between physical multimorbidity status (disease count) and depression and anxiety at baseline using logistic regression. Secondly, we assessed cross-sectional associations between physical multimorbidity patterns and depression and anxiety at baseline using logistic regression.

### 2.9 Role of the funding source

The funder had no role in the design and conduct of the study; collection, management, analysis, and interpretation of the data; preparation, review, or approval of the manuscript; and decision to submit the manuscript for publication.

## 3 Results

### 3.1 Sample characteristics

**Figure 1:**
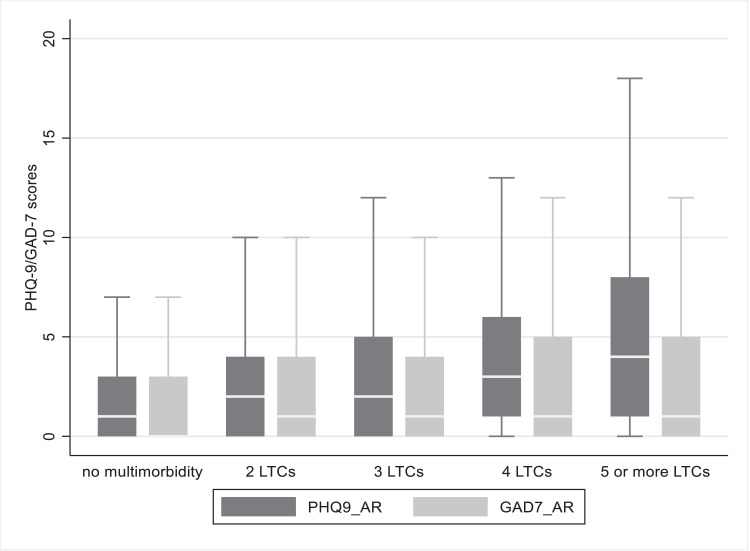
PHQ-9 and GAD-7 scores at follow-up by baseline physical multimorbidity status. Boxes represent 50% of the data, and the error bars represent the remainder. The horizontal lines indicate median values.

The overall sample comprised all participants who completed the PHQ-9 depression measure at follow-up (n = 154,367). Table 1 describes baseline characteristics for the overall sample by physical multimorbidity status. 43,838 (28.4%) participants had multimorbid long-term conditions. Participants with more complex physical multimorbidity (higher number of coexistent disorders) were more likely to be older, to live in socially deprived areas, and to have higher BMI values. Alcohol intake and sedentary behaviour were also more prevalent among those with more complex physical multimorbidity. The likelihood of being an ex- or current smoker was highest in those with four coexistent physical conditions relative to those with no or fewer coexistent physical conditions. Levels of CRP indicated that chronic inflammation was elevated among people with more complex physical multimorbidity. Likewise, the prevalence of depression and anxiety at baseline was greatest among people with more complex physical multimorbidity. Differences in depression and anxiety at follow-up according to physical multimorbidity status are illustrated in Fig. 1.

**Table 1:** Sample characteristics by physical multimorbidity status

	Overall (n=154,367)	No multimorbidity (n=110,529, 71.6%)	Two LTCs (n=26,750, 17.3%)	Three LTCs (n=10,996, 7.1%)	Four LTCS (n=4003, 2.6%)	Five or more LTCs (n=2089, 1.4%)
	*Mean±SD or N(%)*	*Mean±SD or N(%)*	*Mean±SD or N(%)*	*Mean±SD or N(%)*	*Mean±SD or N(%)*	*Mean±SD or N(%)*
Age *(median (IQR))*	57 (50-62)	55 (49-61)	59 (53-63)	61 (55-64)	61 (56-65)	61 (56-65)
Female	87,210 (56.5)	62,471 (56.5)	15,039 (56.2)	6201 (56.4)	2279 (56.9)	1220 (58.4)
Deprivation*	-1.71±2.83	-1.74±2.82	-1.73±2.80	-1.61±2.88	-1.43±2.99	-1.05±3.12
Education level						
*High*	57,300 (37.1)	40,907 (37.0)	10,014 (37.4)	4095 (37.2)	1525 (38.1)	759 (36.3)
*Intermediate*	29,537 (19.1)	21,472 (19.4)	4908 (18.3)	2035 (18.5)	743 (18.6)	379 (18.1)
*Low*	67,530 (43.8)	48,150 (43.6)	11,828 (44.3)	4866 (44.3)	1735 (43.3)	951 (45.5)
Employment status						
*Employed*	45,998 (29.8)	28,037 (25.4)	10,149 (37.9)	4874 (44.3)	1879 (46.9)	1059 (50.7)
*Retired*	98,879 (64.1)	76,260 (69.0)	14,970 (55.3)	5271 (47.9)	1698 (42.4)	680 (33.5)
*Unemployed*⁎⁎	9490 (6.1)	6232 (5.6)	1631 (6.1)	851 (7.7)	426 (10.6)	350 (16.8)
Ethnicity						
*White*	149,924 (97.1)	107,192 (97.0)	26,057 (97.4)	10,746 (97.7)	3900 (97.4)	2029 (97.1)
Ethnic minority group	4443 (2.9)	3337 (3.0)	693 (2.6)	250 (2.3)	103 (2.6)	60 (2.9)
BMI *(kg/m^2^)*	26.77±4.55	26.23±4.17	27.66±4.85	28.49±5.26	29.26±5.55	30.45±6.17
Smoking status						
*Past/current smoker*	65,669 (42.5)	44,667 (40.4)	12,328 (46.1)	5424 (49.3)	2091 (52.2)	1159 (55.5)
*Never smoked*	88,698 (57.5)	65,862 (59.6)	14,422 (53.9)	5572 (50.7)	1912 (47.8)	930 (44.5)
Alcohol intake *(units per week*)	21.08±16.94	20.82±16.70	21.65±17.55	21.77±17.62	21.92±16.69	22.31±17.77
Physical activity						
*None*	1732 (1.1)	928 (0.8)	360 (1.3)	232 (2.1)	103 (2.6)	109 (5.2)
*Low*	27,129 (17.6)	18,379 (16.6)	5026 (18.8)	2258 (20.5)	935 (23.3)	531 (25.4)
*Moderate*	64,704 (41.9)	46,129 (41.7)	11,392 (42.6)	4655 (42.3)	1685 (42.1)	843 (40.3)
*Vigorous*	60,802 (39.4)	45,093 (40.8)	9972 (37.3)	3851 (35.0)	1280 (32.0)	606 (29.0)
Baseline CRP level (mg/L)	2.32±3.94	2.06±3.52	2.70±4.45	3.14±4.95	3.52±5.24	4.39±6.20
Baseline antidepressant use	8068 (5.2)	4291 (3.9)	1765 (6.6)	1056 (9.6)	537 (13.4)	419 (20.1)
Baseline depression						
*Depression*	18,974 (12.3)	12,169 (11.0)	3679 (13.7)	1830 (16.6)	775 (19.4)	521 (24.9)
*No depression*	135,393 (87.7)	98,360 (89.0)	23,071 (86.3)	9166 (83.4)	3228 (80.6)	1568 (75.1)
PHQ-9 follow-up	2.76±3.70	2.51±3.43	3.04±3.88	3.63±4.40	4.31±4.89	5.25±5.34
PHQ-9 depression						
*Depression*	8888 (5.8)	5175 (4.7)	1792 (6.7)	1050 (9.55)	498 (12.4)	373 (17.9)
*No depression*	145,479 (94.2)	105,354 (95.3)	24,958 (93.3)	9946 (90.4)	3505 (87.6)	1716 (82.1)
Baseline anxiety						
*Anxiety*	19,494 (12.6)	11,924 (10.8)	3966 (14.8)	2057 (18.7)	910 (22.7)	637 (30.5)
*No anxiety*	134,873 (87.4)	98,605 (89.2)	22,784 (85.2)	8939 (81.3)	3093 (77.3)	1452 (69.5)
GAD-7 follow-up	2.12±3.35	1.98±3.18	2.25±3.48	2.57±3.86	2.98±4.17	3.47±4.64
GAD-7 anxiety						
*Anxiety*	6500 (4.2)	4037 (3.6)	1238 (4.6)	694 (6.3)	314 (7.8)	217 (10.4)
*No anxiety*	147,867 (95.8)	106,492 (96.4)	25,512 (95.4)	10,302 (93.7)	3689 (92.2)	1872 (89.6)
Follow-up duration *(years)*	7.60±0.87	7.61±0.88	7.59±0.86	7.57±0.85	7.55±0.84	7.55±0.85

BMI=body mass index; GAD=Generalised Anxiety Disorder; IQR=interquartile range; LTC=long-term conditions; PHQ=Patient Health Questionnaire

⁎ Deprivation was measured using the Townsend Deprivation Index. Positive values indicate high material deprivation, whereas negative values indicate relative affluence

⁎⁎ Unemployed includes carers, those in voluntary work, the long-term sick or disabled, and full-time students

### 3.2 Prospective associations between physical multimorbidity status and common mental health disorders

Prospective associations between physical multimorbidity status at baseline and both depression and anxiety at follow-up are presented in Table 2. After adjusting for *a priori* covariates including baseline anxiety, we observed a clear dose-response association between physical multimorbidity status and new onset depression at follow-up. We found that the strength of the association with new onset depression at follow-up ranged from 1.42 (95%CI=1.32 to 1.53) for two physical conditions to 2.89 (95%CI = 2.42 to 3.45) for five or more physical conditions. A similar dose response relationship was observed for incident anxiety.

**Table 2:** Prospective associations between physical multimorbidity and depression and anxiety at follow-up

Depression						
Participants with depression at baseline, outcome: sustained depression (n=18,974)						
	Unadjusted		Age and sex adjusted		Fully adjusted*	
	*OR (95% CI)*	*p value*	*OR (95% CI)*	*p value*	*OR (95% CI)*	*p value*
Physical multimorbidity						
*No multimorbidity*	Reference		Reference		Reference	
*Two conditions*	1.31 (1.19 to 1.44)	<0.001	1.55 (1.40 to 1.70)	<0.001	1.26 (1.14 to 1.40)	<0.001
*Three conditions*	1.80 (1.61 to 2.02)	<0.001	2.27 (2.02 to 2.56)	<0.001	1.64 (1.44 to 1.86)	<0.001
*Four conditions*	2.21 (1.88 to 2.60)	<0.001	2.89 (2.45 to 3.42)	<0.001	1.81 (1.52 to 2.17)	<0.001
*≥ Five conditions*	3.04 (2.53 to 3.66)	<0.001	4.22 (3.48 to 5.10)	<0.001	2.21 (1.79 to 2.72)	<0.001

Participants with no depression at baseline, outcome: incident depression (n=135,393)						
	Unadjusted		Age and sex adjusted		Fully adjusted*	
	*OR (95% CI)*	*p value*	*OR (95% CI)*	*p value*	*OR (95% CI)*	*p value*
Physical multimorbidity						
*No multimorbidity*	Reference		Reference		Reference	
*Two conditions*	1.41 (1.32 to 1.52)	<0.001	1.75 (1.63 to 1.88)	<0.001	1.41 (1.32 to 1.53)	<0.001
*Three conditions*	2.00 (1.82 to 2.19)	<0.001	2.75 (2.50 to 3.02)	<0.001	1.94 (1.76 to 2.14)	<0.001
*Four conditions*	2.66 (2.34 to 3.04)	<0.001	3.87 (3.39 to 4.42)	<0.001	2.38 (2.07 to 2.74)	<0.001
*≥ Five conditions*	3.86 (3.29 to 4.52)	<0.001	5.93 (5.04 to 6.98)	<0.001	2.89 (2.42 to 3.45)	<0.001

Anxiety						
Participants with anxiety at baseline, outcome: sustained anxiety (n=19,494)						
	Unadjusted		Age and sex adjusted		Fully adjusted*	
	*OR (95% CI)*	*p value*	*OR (95% CI)*	*p value*	*OR (95% CI)*	*p value*
Physical multimorbidity						
*No multimorbidity*	Reference		Reference		Reference	
*Two conditions*	1.23 (1.11 to 1.37)	<0.001	1.40 (1.26 to 1.55)	<0.001	1.31 (1.17 to 1.46)	<0.001
*Three conditions*	1.47 (1.29 to 1.67)	<0.001	1.75 (1.54 to 2.00)	<0.001	1.53 (1.34 to 1.76)	<0.001
*Four conditions*	1.74 (1.46 to 2.07)	<0.001	2.13 (1.78 to 2.54)	<0.001	1.75 (1.45 to 2.11)	<0.001
*≥ Five conditions*	1.93 (1.58 to 2.35)	<0.001	2.52 (2.05 to 3.08)	<0.001	1.86 (1.50 to 2.31)	<0.001

Participants with no anxiety at baseline, outcome: incident anxiety (n=134,873)						
	Unadjusted		Age and sex adjusted		Fully adjusted*	
	*OR (95% CI)*	*p value*	*OR (95% CI)*	*p value*	*OR (95% CI)*	*p value*
Physical multimorbidity						
*No multimorbidity*	Reference		Reference		Reference	
*Two conditions*	1.11 (1.02 to 1.19)	0.019	1.31 (1.20 to 1.43)	<0.001	1.21 (1.11 to 1.33)	<0.001
*Three conditions*	1.50 (1.34 to 1.68)	<0.001	1.92 (1.71 to 2.16)	<0.001	1.70 (1.51 to 1.91)	<0.001
*Four conditions*	1.74 (1.46 to 2.07)	<0.001	2.31 (1.93 to 2.75)	<0.001	1.92 (1.60 to 2.30)	<0.001
*≥ Five conditions*	2.26 (1.81 to 2.83)	<0.001	3.11 (2.48 to 3.90)	<0.001	2.31 (1.83 to 2.92)	<0.001

⁎ Covariates: Age, sex, deprivation, education level, employment status, ethnicity, BMI, smoking status, alcohol intake, physical activity, CRP level, follow-up duration, baseline antidepressant use, baseline anxiety (for depression analysis), baseline depression (for anxiety analysis)

In participants who had depression at baseline, fully adjusted models also revealed a dose-response association between physical multimorbidity status and persistent depression at follow-up with the strength of the association ranging from 1.26 (95%CI = 1.14 to 1.40) for two conditions and 2.21 (95%CI = 1.79 to 2.72) for five or more conditions. The same dose-response pattern emerged for persistent anxiety.

### 3.3 Physical multimorbidity patterns at baseline

In the EFA, 13 factors emerged with 77.2% of the variance explained. Rotated factor loadings are provided in Supplementary Material; Table S3. The KMO statistic of 0.61 indicated acceptable sampling adequacy and Bartlett's Test of Sphericity was significant (*p* < 0.001) indicating that factor analysis could be applied to the data. The 13 disease patterns are described in Table 3. Of the 43,838 participants with multimorbidity, 31,173 participants had undefined multimorbidity meaning that 12,665 fell into one or more of the 13 disease patterns that emerged. Disease patterns that included at least 1% of the participants with multimorbidity were included in the main analyses. Five patterns met this criterion: A cardiometabolic pattern (CHD, diabetes, hypertension), a respiratory pattern (asthma, bronchiectasis, COPD), a cardio/cerebrovascular pattern (atrial fibrillation, CHD, stroke/TIA), a reproductive conditions pattern (endometriosis, prostate conditions), and a pain/gastrointestinal pattern (dyspepsia, IBS, painful conditions). Participants were assigned to a multimorbidity pattern if they had two or more of the conditions included in the pattern. Any male participant with multimorbidity which included prostate conditions and any female participant with multimorbidity which included endometriosis were included in the reproductive conditions pattern. Sample characteristics for each multimorbidity cluster are provided in Supplementary Material; Table S5.

**Table 3:** Multimorbidity patterns identified using EFA. Patterns highlighted in bold were included in the main analyses as the number of participants with multimorbidity (n=43,838, 28.4%) who were included in the pattern was greater than 1%

Pattern	Eigenvalue	Long-term conditions	No. of participants (%)
Pattern 1	4.43	CFS, CKD, epilepsy, Parkinson's disease	23 (0.05)
**Pattern 2** ***Cardiometabolic***	**3.19**	**CHD, diabetes, hypertension**	**5101 (11.6)**
Pattern 3	2.46	Hepatitis, liver disease	27 (0.06)
Pattern 4	2.11	Dementia, glaucoma, Meniere's disease, migraine	77 (0.2)
**Pattern 5** ***Respiratory***	**1.84**	**Asthma, bronchiectasis, COPD**	**727 (1.6)**
Pattern 6	1.48	Asthma, PCOS, PVD	94 (0.2)
**Pattern 7** ***Cardio/cerebrovascular***	**1.43**	**Atrial fibrillation, CHD, stroke/TIA**	**621 (1.4)**
Pattern 8* Reproductive	**1.35**	**Endometriosis, prostate conditions**	**3055 (7.0)**
Pattern 9	1.23	Heart failure, MS	0 (0.0)
**Pattern 10** ***Pain/gastrointestinal***	**1.18**	**Dyspepsia, IBS, painful conditions**	**4999 (11.4)**
Pattern 11	1.12	Pernicious anaemia, thyroid problems	107 (0.2)
Pattern 12	1.05	Cancer, osteoporosis	330 (0.7)
Pattern 13	1.01	Connective tissue disorders, psoriasis/eczema	283 (0.6)

CFS=chronic fatigue syndrome; CKD=chronic kidney disease; COPD=chronic obstructive pulmonary disorder; IBS=irritable bowel syndrome; MS=multiple sclerosis; PCOS=polycystic ovarian syndrome; PVD=peripheral vascular disease; TIA=transient ischaemic attack

⁎ Any male participant with multimorbidity which comprised prostate conditions was included in this pattern; any female with multimorbidity which comprised endometriosis was included in this pattern Note: Although 12,665 participants fell into one or more of the multimorbidity patterns, the total here of 15,444 participants reflects a certain amount of overlap across patterns. In pattern 9, no participants experienced both conditions constituting the factor. This is likely an artefact of using tetrachoric correlations and an arbitrary factor loading of +/-0.4 to determine groups. It is probable that these two conditions are linked via a third condition that is either unmeasured or included in the analysis but is subthreshold.

### 3.4 Prospective associations between specific physical multimorbidity patterns and common mental health disorders

Table 4 presents the findings for the prospective associations between specific physical multimorbidity patterns at baseline and depression and anxiety at follow-up separately in those with and without depression and anxiety at baseline, respectively. Fully adjusted models revealed that almost all multimorbidity patterns were significant predictors of both incident and persistent CMDs at follow-up, with the exceptions of the reproductive conditions pattern and persistent depression (*p* = 0.349) and the cardio/cerebrovascular pattern and incident anxiety (*p* = 0.566).

**Table 4:** Prospective associations between physical multimorbidity patterns and depression at follow-up

Depression						
Participants with depression at baseline (n=18,974)						
	Unadjusted		Age and sex adjusted		Fully adjusted*	
	*OR (95% CI)*	*p value*	*OR (95% CI)*	*p value*	*OR (95% CI)*	*p value*
Physical multimorbidity pattern						
*No multimorbidity*	Reference		Reference		Reference	
*Undefined multimorbidity*	1.40 (1.29 to 1.53)	<0.001	1.65 (1.51 to 1.80)	<0.001	1.31 (1.19 to 1.44)	<0.001
*Cardiometabolic*	2.40 (2.04 to 2.83)	<0.001	3.33 (2.80 to 3.95)	<0.001	1.85 (1.53 to 2.24)	<0.001
*Respiratory*	2.38 (1.69 to 3.37)	<0.001	3.16 (2.22 to 4.49)	<0.001	1.97 (1.35 to 2.88)	<0.001
*Cardio/cerebrovascular*	3.13 (1.98 to 4.93)	<0.001	1.18 (3.12 to 7.91)	<0.001	2.56 (1.54 to 4.27)	<0.001
*Reproductive*	1.19 (0.94 to 1.52)	0.147	1.50 (1.17 to 1.91)	0.001	1.13 (0.87 to 1.46)	0.349
*Pain/gastrointestinal*	2.41 (2.10 to 2.76)	<0.001	3.00 (2.60 to 3.45)	<0.001	1.92 (1.65 to 2.24)	<0.001

Participants with no depression at baseline (n=135,393)						
	Unadjusted		Age and sex adjusted		Fully adjusted*	
	*OR (95% CI)*	*p value*	*OR (95% CI)*	*p value*	*OR (95% CI)*	*p value*
Physical multimorbidity pattern						
*No multimorbidity*	Reference		Reference		Reference	
*Undefined multimorbidity*	1.58 (1.48 to 1.68)	<0.001	1.96 (1.83 to 2.09)	<0.001	1.54 (1.44 to 1.65)	<0.001
*Cardiometabolic*	2.10 (1.85 to 2.38)	<0.001	3.40 (2.98 to 3.87)	<0.001	1.93 (1.68 to 2.23)	<0.001
*Respiratory*	3.77 (2.91 to 4.90)	<0.001	5.08 (3.89 to 6.62)	<0.001	3.23 (2.44 to 4.27)	<0.001
*Cardio/cerebrovascular*	1.92 (1.35 to 2.73)	<0.001	3.51 (2.45 to 5.02)	<0.001	2.11 (1.45 to 3.07)	<0.001
*Reproductive*	1.64 (1.37 to 1.95)	<0.001	2.44 (2.04 to 2.93)	<0.001	2.04 (1.70 to 2.46)	<0.001
*Pain/gastrointestinal*	2.61 (2.31 to 2.94)	<0.001	3.39 (3.00 to 3.84)	<0.001	2.19 (1.92 to 2.50)	<0.001

Anxiety						
Participants with anxiety at baseline (n=19,494)						
	Unadjusted		Age and sex adjusted		Fully adjusted*	
	*OR (95% CI)*	*p value*	*OR (95% CI)*	*p value*	*OR (95% CI)*	*p value*
Physical multimorbidity pattern						
*No multimorbidity*	Reference		Reference		Reference	
*Undefined multimorbidity*	1.30 (1.18 to 1.43)	<0.001	1.45 (1.31 to 1.59)	<0.001	1.35 (1.22 to 1.50)	<0.001
*Cardiometabolic*	1.60 (1.34 to 1.91)	<0.001	2.13 (1.76 to 2.57)	<0.001	1.79 (1.46 to 2.20)	<0.001
*Respiratory*	1.85 (1.29 to 2.66)	0.001	2.27 (1.57 to 3.27)	<0.001	1.90 (1.30 to 2.78)	0.001
*Cardio/cerebrovascular*	1.37 (0.84 to 2.21)	0.204	1.95 (1.20 to 3.18)	0.007	1.70 (1.03 to 2.83)	0.038
*Reproductive*	1.25 (0.97 to 1.63)	0.089	1.51 (1.16 to 1.97)	0.002	1.34 (1.02 to 1.77)	0.033
*Pain/gastrointestinal*	1.77 (1.52 to 2.06)	<0.001	2.03 (1.74 to 2.37)	<0.001	1.64 (1.39 to 1.93)	<0.001

Participants with no anxiety at baseline (n=134,873)						
	Unadjusted		Age and sex adjusted		Fully adjusted*	
	*OR (95% CI)*	*p value*	*OR (95% CI)*	*p value*	*OR (95% CI)*	*p value*
Physical multimorbidity pattern						
*No multimorbidity*	Reference		Reference		Reference	
*Undefined multimorbidity*	1.27 (1.17 to 1.37)	<0.001	1.48 (1.37 to 1.60)	<0.001	1.35 (1.25 to 1.47)	<0.001
*Cardiometabolic*	1.11 (0.92 to 1.34)	0.262	1.74 (1.44 to 2.10)	<0.001	1.42 (1.16 to 1.72)	<0.001
*Respiratory*	1.73 (1.14 to 2.61)	0.009	2.15 (1.42 to 3.26)	<0.001	1.75 (1.15 to 2.66)	0.009
*Cardio/cerebrovascular*	0.76 (0.41 to 1.42)	0.396	1.37 (0.73 to 2.57)	0.329	1.20 (0.64 to 2.27)	0.566
*Reproductive*	1.10 (0.87 to 1.39)	0.410	1.62 (1.28 to 2.05)	<0.001	1.47 (1.16 to 1.86)	0.002
*Pain/gastrointestinal*	1.91 (1.64 to 2.22)	<0.001	2.30 (1.97 to 2.69)	<0.001	1.90 (1.62 to 2.23)	<0.001

⁎ Covariates: Age, sex, deprivation, education level, employment status, ethnicity, BMI, smoking status, alcohol intake, physical activity, CRP level, follow-up duration, baseline antidepressant use, baseline anxiety (for depression analysis), baseline depression (for anxiety analysis)

In terms of incident depression and anxiety, the odds ratios revealed that the respiratory pattern (depression: aOR = 3.23, 95%CI 2.44 to 4.27, anxiety: aOR = 1.75, 95%CI 1.15 to 2.66), and the pain/gastrointestinal pattern (depression: aOR = 2.19, 95%CI 1.92 to 2.50, anxiety: aOR = 1.90, 95%CI 1.62 to 2.23) were most strongly associated with CMD outcomes at follow-up. The respiratory pattern was also strongly associated with both persistent depression (aOR = 1.97, 95%CI 1.35 to 2.88) and anxiety (aOR = 1.90, 95%CI = 1.30 to 2.78). The cardio/cerebrovascular pattern was a strong predictor of persistent depression (aOR = 2.56, 95%CI 1.54 to 4.27) and the cardiometabolic pattern was a strong predictor of persistent anxiety (aOR=1.79, 95%CI = 1.46 to 2.20).

### 3.5 Sensitivity analyses

Three sensitivity analyses were performed. First, comparisons between those who completed the mental health follow-up questionnaire (the analytical sample) and those who did not complete the questionnaire (remainder) showed that the analytical sample differed significantly from the remainder. Results are presented in Supplementary Material; Table S2. The analytical sample were younger, less deprived, and less likely to be from an ethnic minority group. They also were less likely to be current or past smokers, drank less alcohol, and were less sedentary than those who did not complete the follow-up questionnaire. Participants in the analytical sample were less depressed and less anxious at baseline and were less likely to have physical multimorbidity.

Second, cross-sectional associations between physical multimorbidity status and common mental health disorders at baseline that included the total population (Supplementary Material; Table S4) validated the dose-response relationship between the number of co-existing disorders with the likelihood of anxiety. However, the cross-sectional associations between physical multimorbidity status and depression were more mixed indicating that other factors might be at play. Third, the multimorbidity pattern most strongly associated with depression was the pain/gastrointestinal pattern, while for anxiety it was the respiratory pattern (Supplementary Material: Table S6).

## 4 Discussion

The current study examined prospective associations between physical multimorbidity patterns and common mental health disorders in middle-aged adults from the UK Biobank. Physical multimorbidity status was associated with both incident depression and anxiety in a dose-response manner, in that as the number of physical conditions increased so did the likelihood of incident poor mental health. Similar results emerged for persistent depression and anxiety, but the odds ratios suggested that associations were somewhat stronger for incident CMDs. We identified five patterns of multimorbidity, most of which associated to some degree with future depression and anxiety. There was variation in how strongly these multimorbidity patterns associated with incident and persistent CMDs which implies that certain patterns of diseases might increase the likelihood of CMD symptoms more than others. The respiratory and pain/gastrointestinal patterns consistently emerged as the strongest predictors of incident depression and anxiety among middle-aged UK adults.

The observed associations we describe between physical multimorbidity status and future incident depression corroborate the prospective findings of previous studies [10,11,13,[40], [41], [42]], particularly those studies that report a dose-response relationship [19]. However, to the best of our knowledge, this is the first study to show that physical multimorbidity can lead to future anxiety specifically. Of interest in the current study is that associations between physical multimorbidity and incident depression and anxiety were stronger than associations with persistent depression and anxiety. Previous studies have found that physical health was a strong predictor of incident depression, whereas findings relating to persistent depression have been more mixed [43]. This suggests that other factors might be more important in the persistence of CMDs, such as social isolation [43,44].

In the current study, we identified five patterns of physical multimorbidity which are generally similar to those reported in previous studies. The most commonly identified disease pattern in previous studies has comprised cardiovascular and metabolic disorders [19,[45], [46], [47], [48], [49], [50]], which is unsurprising considering the shared aetiology of these conditions. In the current study, we identified two cardiovascular patterns which might imply that there are different types of cardiovascular multimorbidity that are influenced by different factors. For example, those in the cardiometabolic pattern were younger, less deprived, and more likely to be of from a minority ethnic group compared to those in the cardio/cerebrovascular pattern. More work is needed to understand the factors involved in cardiovascular multimorbidity. The respiratory pattern identified in the current study has also been reported previously [19,47,[49], [50], [51]]. Asthma is known to be a risk factor for the development of COPD [52], and both conditions are known to overlap, particularly in older adults [53]. The pain/gastrointestinal cluster we observed included conditions such as back pain, arthritis, and IBS. Several earlier studies also identified disease clusters which comprised musculoskeletal pain, arthritis, and gastrointestinal conditions [19,45,50]. Potential common mechanisms underlying this group of conditions includes the use of pain medications [54], difficulty engaging in physical activity [55], and potential changes in gut bacteria [56]. The reproductive conditions pattern that emerged in the current study comprised men with multimorbidity that included prostate conditions and women with multimorbidity that included endometriosis. It is plausible that these sex-specific conditions share common hormonal pathways, but more work is needed to understand this.

The findings of the current study are broadly in line with the only previous study to adopt an exploratory approach to determine physical multimorbidity clusters, and the associations between these clusters and future depression. Like Yao and colleagues [19], we found that most physical multimorbidity clusters, including the undefined multimorbidity cluster, were associated with future common mental health disorders to some extent which indicates that physical multimorbidity can affect mental health regardless of the conditions involved. It comes as no surprise that physical multimorbidity might result in depression and/or anxiety due to mediating factors such as chronic pain [57], frailty [58,59], symptom burden [60], functional impairment [61], and reduced quality of life [4,62]. There are also potential direct biological pathways through which physical multimorbidity might lead to common mental health disorders. Inflammation is known to play a causal role in depression [63] and most physical long-term conditions are characterised by a high inflammatory burden. Dysregulation of the hypothalamic-pituitary-adrenal axis is consistently reported in patients with depression, and there is evidence that the presence of physical illness might play a role in this [64]. Furthermore, there is evidence that complex treatment of physical disease and polypharmacy might play a role in the development of depression in multimorbid patients [65].

Understanding the extent to which specific combinations of physical illness increase the likelihood of depression and anxiety might allow us to identify specific mediating factors and develop targeted interventions for patient groups. Yao and colleagues reported that the respiratory disease pattern and the arthritic-digestive-visual disease pattern were the strongest predictors of future depressive symptoms in middle-aged and older Chinese adults [19]. In the current study, we replicated these findings by showing that the respiratory disease and the pain/gastrointestinal disease patterns were the strongest predictors of future incident common mental health problems in middle-aged adults in the UK. What this suggests is that there may be mechanisms specific to these multimorbid conditions that increase the likelihood of developing depression and anxiety. For example, the degree of airflow limitation in COPD has been found to be associated with anxiety and depression [66]. Sleep problems, such as obstructive sleep apnoea, are common in chronic respiratory disease [67] and are also known to play a role in depression [68]. Both asthma and COPD are frequently treated with high doses of corticosteroids, the long-term use of which is known to induce depressive symptoms [69]. In terms of pain/gastrointestinal multimorbidity, the chronic pain [70], sleep disturbances, and functional impairment [71] associated with conditions such as back problems, arthritis, and IBS are likely factors in the development of depression and anxiety in these patients. Future research needs to focus on delineating the specific mechanisms linking certain clusters of physical multimorbidity with depression and anxiety. This would facilitate the development of patient-oriented prevention and treatment strategies for common mental health disorders in patients with physical multimorbidity.

### 4.1 Strengths and limitations

In addition to the large sample size, the use of UK Biobank data allowed for the inclusion of a very broad number of long-term physical conditions defined using a combination of both self-report and hospital episode data. The UK Biobank also allowed us to adjust for a considerable number of factors known to affect both physical multimorbidity status and depression or anxiety. The use of standardised psychometrics with established clinical cut-offs for the measurement of depression and anxiety is a further strength of the current study. The use of EFA to identify specific clusters of physical multimorbidity allowed physical conditions to cross-cluster which provided a more realistic view of how conditions group together.

Several limitations need consideration. A major limitation associated with observational studies is difficulty establishing causality and residual confounding. Although we controlled for a significant number of relevant confounders, we cannot reject the possibility of unmeasured confounders that might bias our results. The use of self-report for the measurement of physical long-term conditions is susceptible to bias and inaccuracies. Furthermore, research has shown that people with depression demonstrate a recall bias for negative information [72], which may have affected self-report in the current study. However, we also used linked hospital admissions data to define the physical long-term conditions which would have somewhat reduced the bias associated with self-report. We were not able to account for disease severity and changes in multimorbidity over time. Assessing multimorbidity trajectories, particularly in those with undefined multimorbidity, poses a challenge for future research. Based on previous research [49], it is likely that considerable changes in physical multimorbidity status occurred in the time between the baseline assessment and the mental health follow-up which we could not account for in the current study. Moreover, due to the nature of the data, we were unable to account for changes in the relevant covariates over time.

Although we included a comprehensive list of long-term physical conditions in the current study, it was not possible to include several important conditions known to influence mental health, such as blindness and hearing loss [73]. We used comprehensive measures of depression and anxiety at baseline, but depression and anxiety were measured at follow-up using the PHQ-9 and the GAD-7, respectively. This inconsistency between measurement of depression and anxiety across timepoints might introduce some discrepancies in caseness at baseline and follow-up. Moreover, it is worth noting that both the PHQ-9 and GAD-7 are not measures of clinical depression and anxiety respectively, although the PHQ-9 has shown suitable psychometric properties when compared to structured clinical interviews [74] and the GAD-7 has demonstrated good sensitivity and specificity for the diagnosis of the most common anxiety disorders in primary care [32]. In the current study depression and anxiety were measured once over the follow-up period meaning that we were unable to account for the complex clinical courses that common mental health disorders can take. It is also important to note that the analyses of the outcomes of these patterns are only for a subpopulation where these patterns could be identified and hence the interpretation cannot be extended to the whole participating population. These analyses provide, nevertheless, a comparative framework for evaluating how future changes in the patterning of physical multimorbidity might affect the incidence of common mental health disorders at population level or by specific at-risk sub-groups (e.g., elderly, socially deprived, ethnic minority).

The UK Biobank comprises middle-aged participants which is one of the strengths of the current study as most of the previous evidence was based on clinical and/or older populations where common mental health disorders are less well captured [9], [10], [11], [12], [13], [14]. However, the generalisability of the results is limited to this population subgroup. The UK Biobank cohort is also known to differ from the general UK population in terms of demographic (more female, less deprived) and health (less smoking, fewer self-reported health conditions) factors which will also affect the generalisability of results [75]. Moreover, the sample used in the current study only comprised UK Biobank participants who completed a mental health follow-up questionnaire. The study sample differed significantly on sociodemographic, behavioural, and clinical factors appearing to be both mentally and physically ‘healthier’ when compared to those not included in the analysis. Although this introduces significant bias and further compromises the generalisability of results, the higher rates of baseline depression, anxiety, and physical multimorbidity seen in the excluded participants suggests that even stronger associations between physical multimorbidity and future CMDs would emerge if these data had been collected.

### 4.2 Conclusions and implications

The current study showed that physical multimorbidity is associated with both incident and persistent depression and anxiety in middle-aged UK adults. Moreover, it was the first UK-based study to identify patterns of physical multimorbidity in middle-age and assess the extent to which these patterns associated with future likelihood of common mental health disorders. Results indicated that the respiratory disease pattern and the pain/gastrointestinal pattern were the strongest predictors of incident depression and anxiety. These findings might have significant implications for the implementation of integrated mental and physical healthcare, highlighting the extent to which different clusters of physical disease increase the likelihood of poor mental health. Understanding the specific mechanisms through which specific disease clusters affect mental health will help facilitate the development of targeted preventative interventions and treatment for people with physical multimorbidity.

## Contributors

AR was responsible for the conceptualisation, formal analysis, and writing of the original draft of the manuscript. JAT contributed to the formal analysis and writing (review and editing) of the manuscript. MP, DA, JDM, SH, RS, MH, and AD contributed to the writing (review and editing) of the manuscript. AD also was responsible for data curation and funding acquisition.

## Data sharing

Data from UK Biobank are available to all researchers upon making an application. The study was conducted under UK Biobank project number 34554.

## Declaration of Competing Interests

This study represents independent research part-funded by the National Institute for Health Research (NIHR) Biomedical Research Centre at South London and Maudsley NHS Foundation Trust and King's College London. The views expressed are those of the author(s) and not necessarily those of the NHS, the NIHR or the Department of Health and Social Care.

AR, JAT, DA, JDM, AD, MP have nothing to disclose.

RS reports grants from Janssen, GSK, and Takeda outside the submitted work. SH reports grants from NIHR, grants from ESRC, grants from Wellcome Trust, grants from MRC, grants from Guy's & St. Thomas's Charity, outside the submitted work; SH is a member of the following: Expert Review Group (ERG) of the UK Prevention Research Partnership (UKPRP) Ethnic inequalities in health care among people with multiple conditions (University of Sussex) – Advisory Board NHS Race and Health Observatory, Co-Chair Academic Reference Group and Board Member The Royal Foundation - Mental Health Research Group NHS England and NHS Improvement - The Mental Health Equalities Data Quality and Research Subgroup NHS England and NHS Improvement - Patient and Carers Race Equalities Framework [PCREF] Steering Group NHS England and NHS Improvement - Advancing Mental Health Equalities Taskforce Health Education England - Mental Health Workforce Equalities Subgroup Maudsley Learning - Maudsley Learning Advisory Board South London and Maudsley NHS Foundation Trust (SLaM) - Independent Advisory Groups, the SLaM Partnership Group Lambeth Public Health - Serious Youth Violence Public Health Task and Finish Group Thrive London - Thrive London Advisory Board Black Thrive - Black Thrive Advisory Board NHS England and NHS Improvement - The Mental Health Workforce Equalities Subgroup Commissions: Welsh Government's Race Equality Plan; contribution to the evidence review for Health and Social Care and Employment and Income policy areas.

## Appendix Supplementary materials

Image, application 1
