# Supplementary material for: Associations between physical multimorbidity patterns and common mental health disorders in middle-aged adults: A prospective analysis using data from the UK Biobank
Source: Lancet Reg Health Eur. 2021 Jun 22;8:100149. doi: 10.1016/j.lanepe.2021.100149 (PMC8447568; doi:10.1016/j.lanepe.2021.100149)
Supplement: Supplementary file 1 [file mmc1.docx]

| **Table S1.** UK Biobank codes for physical long-term conditions | |  |
| --- | --- | --- |
| Condition | UK Biobank coding (data field 20002) | Hospital Episode Statistics (ICD-10 codes) |
| Asthma | 1111 Asthma | J45 Asthma |
| Atrial fibrillation | 1471 Atrial fibrillation | I48 Atrial fibrillation and flutter  I49 Other cardiac arrythmias |
| Bronchiectasis | 1114 Bronchiectasis | J47 Bronchiectasis |
| Cancer | UK Biobank data field 2453 | C17 Malignant neoplasm of small intestine  C18 Malignant neoplasm of colon  C19 Malignant neoplasm of rectosigmoid junction  C20 Malignant neoplasm of rectum  C21 Malignant neoplasm of anus and anal canal  C16 Malignant neoplasm of stomach  C15 Malignant neoplasm of oesophagus  C22 Malignant neoplasm of liver and intrahepatic bile ducts  C25 Malignant neoplasm of pancreas  C50 Malignant neoplasm of breast  C34 Malignant neoplasm of bronchus and lung  C40 Malignant neoplasm of bone and articular cartilage of limbs  C41 Malignant neoplasm of bone and articular cartilage of other and unspecified sites  C43 Malignant melanoma of skin  C71 Malignant neoplasm of brain  C53 Malignant neoplasm of cervix uteri  C55 Malignant neoplasm of uterus, part unspecified  C56 Malignant neoplasm of ovary  C61 Malignant neoplasm of prostate  C64 Malignant neoplasm of kidney, except renal pelvis  C80 Malignant neoplasm without specification of site  C81 Lymphocyte-rich classical Hodgkin lymphoma  C82 Follicular lymphoma  C83 Non-follicular lymphoma  C84 Mature T/NK-cell lymphomas  C85 Other specified and unspecified types of non-Hodgkin lymphoma  C86 Other specified types of T/NK-cell lymphoma  C88 Malignant immunoproliferative diseases and certain other B-cell lymphomas  C90 Multiple myeloma and malignant plasma cell neoplasms  C91 Lymphoid leukaemia  C92 Myeloid leukaemia  C93 Monocytic leukaemia  C94 Other leukaemias of specified cell type  C95 Leukaemia of unspecified cell type  C96 Other and unspecified malignant neoplasms of lymphoid, hematopoietic and related tissue  C00 Malignant neoplasm of lip  C01 Malignant neoplasm of base of tongue  C02 Malignant neoplasm of other and unspecified parts of tongue  C03 Malignant neoplasm of gum  C04 Malignant neoplasm of floor of mouth  C05 Malignant neoplasm of palate  C06 Malignant neoplasm of other and unspecified parts of mouth |
| Chronic fatigue syndrome (CFS) | 1482 Chronic fatigue syndrome | R53.82 Chronic fatigue, unspecified |
| Chronic kidney disease (CKD) | 1192 Renal/kidney failure  1193 Renal failure requiring dialysis  1194 Renal failure not requiring dialysis  1427 Polycystic kidney  1519 Kidney nephropathy  1520 IGA nephropathy  1607 Diabetic nephropathy | N17 Acute kidney failure  N18 Chronic kidney disease (CKD)  N19 Unspecified kidney failure  N00 Acute nephritic syndrome  N01 Rapidly progressive nephritic syndrome  N03 Chronic nephritic syndrome  N04 Nephrotic syndrome  N05 Unspecified nephritic syndrome  N08 Glomerular disorders in diseases classified elsewhere  E08.22 Diabetes mellitus due to underlying condition with diabetic chronic kidney disease  E09.22 Drug or chemical induced diabetes mellitus with diabetic chronic kidney disease  E10.22 Type 1 diabetes mellitus with diabetic chronic kidney disease  E11.22 Type 2 diabetes mellitus with diabetic chronic kidney disease  E13.22 Other specified diabetes mellitus with diabetic chronic kidney disease  I12 Hypertensive chronic kidney disease  I13 Hypertensive heart and chronic kidney disease |
| Chronic obstructive pulmonary disorder (COPD) | 1112 Chronic obstructive airways disease/COPD  1113 Emphysema/chronic bronchitis  1472 Emphysema | J44 Other chronic obstructive pulmonary disease  J43 Emphysema  J42 Unspecified chronic bronchitis  J41 Simple and mucopurulent chronic bronchitis  J40 Bronchitis, not specified as acute or chronic |
| Chronic sinusitis | 1416 Chronic sinusitis | J32 Chronic sinustitis |
| Connective tissue disorders | 1322 Myositis/myopathy  1373 Connective tissue disorder  1377 Polymyalgia rheumatica  1381 Systemic lupus erythematosis/SLE  1382 Sjogren’s syndrome/sicca syndrome  1383 Dermatopolymyositis  1384 Scleroderma/systemic sclerosis  1456 Malabsorption/coeliac disease  1464 Rheumatoid arthritis  1477 Psoriatic arthropathy  1480 Dermatomyositis  1481 Polymyositis | K90 Intestinal malabsorption/coeliac disease  M05 Rheumatoid arthritis  M06 Other rheumatoid arthritis  M07 Psoriatic and enteropathic arthropathies  M08 Juvenile arthritis  M30 Polyarteritis nodosa and related conditions  M31 Other necrotizing vasculopathies  M32 Systemic lupus erythematosus  M33 Dermatopolymyositis  M34 Systemic sclerosis  M35 Other systemic involvement of connective tissue  M36 Systemic disorders of connective tissue in diseases classified elsewhere  M60 Myositis |
| Coronary heart disease (CHD) | 1074 Angina  1075 Heart attack/myocardial infarction | I20 Angina pectoris  I21 Acute myocardial infarction  I22 Subsequent myocardial infarction  I23 Certain current complications following acute myocardial infarction  I24 Other acute ischaemic heart diseases  I25 Chronic ischaemic heart disease |
| Dementia | 1263 Dementia/alzheimers/cognitive impairment | F01 Vascular dementia  F02 Dementia in other diseases classified elsewhere  F03 Unspecified dementia  G30 Alzheimer disease  G31.83 Dementia with Lewy bodies  G31.0 Circumscribed brain atrophy  G31.1 Senile degeneration of brain, not elsewhere classified  G31.01 Pick’s disease  G31.09 Other frontotemporal dementia |
| Diabetes | 1220 Diabetes  1222 Type 1 diabetes  1223 Type 2 diabetes  1276 Diabetic eye disease  1468 Diabetic neuropathy/ulcers  1607 Diabetic nephropathy | E11 Non-insulin-dependent diabetes mellitus  E10 Insulin-dependent diabetes mellitus  E09 Drug or chemical induced diabetes mellitus  E08 Diabetes mellitus due to underlying condition  E13 Other specified diabetes mellitus  O24.4 Gestational diabetes |
| Diverticular disease | 1458 Diverticular disease/diverticulitis | K57 Diverticular disease of intestine |
| Dyspepsia/ulcer | 1138 Gastro-oesophageal reflux/gastric reflux  1139 Oesophagitis/barretts oesophagus  1142 Gastric/stomach ulcers  1143 Gastritis/gastric erosions  1442 Helicobacter pylori  1457 Duodenal ulcer  1474 Hiatus hernia  1510 Dyspepsia/indigestion | K21 Gastro-oesophageal reflux disease  K22 Other diseases of oesophagus  K25 Gastric ulcer  K26 Duodenal ulcer  K27 Peptic ulcer, site unspecified  K28 Gastrojejunal ulcer  K29 Gastritis and duodenitis  K30 Dyspepsia |
| Endometriosis | 1402 Endometriosis | N80 Endometriosis |
| Epilepsy | 1264 Epilepsy | G40 Epilepsy |
| Glaucoma | 1277 Glaucoma | H40 Glaucoma  H42 Glaucoma in diseases classified elsewhere |
| Heart failure | 1076 Heart failure/pulmonary oedema  1079 Cardiomyopathy  1588 Hypertrophic cardiomyopathy | I46 Cardiac arrest  I50 Heart failure |
| Hepatitis | 1156 Infective/viral hepatitis  1578 hepatitis A  1579 hepatitis B  1580 Hepatitis C  1581 Hepatitis D  1582 Hepatitis E | B15 Acute hepatitis A  B16 Acute hepatitis B  B17 Other acute viral hepatitis  B18 Chronic viral hepatitis  B19 Unspecified viral hepatitis |
| Hypertension | 1065 Hypertension  1072 Essential hypertension | I10 Essential (primary) hypertension  I11 Hypertensive heart disease  I12 Hypertensive renal disease  I13 Hypertensive heart and renal disease  I15 Secondary hypertension |
| Inflammatory bowel disease (IBD) | 1461 Inflammatory bowel disease  1462 Crohn’s disease  1463 Ulcerative colitis | K50 Crohn disease [regional enteritis]  K51 Ulcerative colitis  K52 Other noninfective gastroenteritis and colitis |
| Irritable bowel syndrome (IBS) | 1154 Irritable bowel syndrome | K58 Irritable bowel syndrome |
| Liver disease | 1141 Oesophageal varicies  1157 Non-infective hepatitis  1158 Liver failure/cirrhosis  1506 Primary biliary cirrhosis | K70 Alcoholic liver disease  K71 Toxic liver disease  K72 Hepatic failure, not elsewhere classified  K73 Chronic hepatitis, not elsewhere classified  K74 Fibrosis and cirrhosis of liver  K75 Other inflammatory liver diseases  K76 Other diseases of liver  K77 Liver disorders in diseases classified elsewhere |
| Méniére’s disease | 1421 Méniére disease | H81.0 Méniére disease |
| Migraine | 1265 Migraine | G43 Migraine |
| Multiple sclerosis (MS) | 1261 Multiple sclerosis | G35 Multiple sclerosis |
| Osteoporosis | 1309 Osteoporosis | M80 Osteoporosis with pathological fracture  M81 Osteoporosis without pathological fracture  M82 Osteoporosis in diseases classified elsewhere |
| Painful conditions | 1257 Trapped nerve/compressed nerve  1294 Back problem  1311 Spine arthritis/spondylitis  1312 Prolapsed disc/slipped disc  1313 Ankylosing spondylitis  1436 Headaches (not migraine)  1465 Osteoarthritis  1466 Gout  1476 Sciatica  1478 Cervical spondylosis  1523 Trigeminal neuralgia  1532 Disc problem  1533 Disc degeneration  1534 Back pain  1537 Joint pain  1538 Arthritis  1540 Plantar fasciitis  1541 Carpal tunnel syndrome  1542 Fibromyalgia  1573 Shingles | M45 Ankylosing spondylitis  M46 Other inflammatory spondylopathies  M47 Spondylosis  M48 Other spondylopathies  M49 Spondylopathies in diseases classified elsewhere  M50 Cervical disc disorders  M51 Other intervertebral disc disorders  M53 Other dorsopathies, not elsewhere classified  M54 Dorsalgia  R51 Headache  G44 Other headache syndromes  M10 Gout  M11 Other crystal arthropathies  M12 Other specific arthropathies  M13 Other arthritis  M14 Arthropathies in other diseases classified elsewhere  M72.2 Plantar fasciitis  G50.0 Trigeminal neuralgia  G50.1 Atypical facial pain  G56.0 Carpal tunnel syndrome  M79.7 Fibromyalgia  B02 Herpes zoster (shingles) |
| Parkinson’s disease | 1262 Parkinson’s disease | G20 Parkinson’s disease  G21 Secondary parkinsonism |
| Peripheral vascular disease (PVD) | 1067 Peripheral vascular disease  1087 Leg claudication/intermittent claudication | I700 Atherosclerosis of aorta  I702 Atherosclerosis of arteries of extremities  I708 Atherosclerosis of other arteries  I709 Generalized and unspecified atherosclerosis  I731 Thromboangiitis obliterans [Buerger]  I738 Other specified peripheral vascular diseases  I739 Peripheral vascular disease, unspecified |
| Pernicious anaemia | 1331 Pernicious anaemia | D51.0 Vitamin B12 deficiency anaemia due to intrinsic factor deficiency |
| Polycystic ovarian syndrome (PCOS) | 1350 Polycystic ovarian syndrome | E28.2 Polycystic ovarian syndrome |
| Prostate conditions (not cancer) | 1207 Prostate problem (not cancer)  1396 Enlarged prostate  1516 Benign prostatic hypertrophy | N40 Hyperplasia of prostate  N41 Inflammatory diseases of prostate  N42 Other disorders of prostate |
| Psoriasis/eczema | 1452 Eczema/dermatitis  1453 Psoriasis | L20 Atopic dermatitis  L21 Seborrhoeic dermatitis  L22 Diaper [napkin] dermatitis  L23 Allergic contact dermatitis  L24 Irritant contact dermatitis  L25 Unspecified contact dermatitis  L26 Exfoliative dermatitis  L27 Dermatitis due to substances taken internally  L28 Lichen simplex chronicus and prurigo  L29 Pruritis  L30 Other and unspecified dermatitis  L40 Psoriasis  L41 Parapsoriasis |
| Stroke/transient ischaemic attack (TIA) | 1081 Stroke  1082 Transient ischaemic attack  1086 Subarachnoid haemorrhage  1491 Brain haemorrhage  1583 Ischaemic stroke | I60 Subarachnoid haemorrhage  I61 Intracerebral haemorrhage  I62 Other nontraumatic intracranial haemorrhage  I63 Cerebral infarction  I65 Occlusion and stenosis of precerebral arteries, not resulting in cerebral infarction  I66 Occlusion and stenosis of cerebral arteries, not resulting in cerebral infarction  I67 Other cerebrovascular diseases  I68 Cerebrovascular disorders in diseases classified elsewhere  I69 Sequelae of cerebrovascular disease |
| Thyroid conditions | 1224 Thyroid problem (not cancer)  1225 Hyperthyroidism/thyrotoxicosis  1226 Hypothyroidism/myxoedema  1428 Thyroiditis  1522 Grave’s disease  1610 Thyroid goitre | E00 Congenital iodine-deficiency syndrome  E01 Iodine-deficiency-related thyroid disorders and allied conditions  E02 Subclinical iodine-deficiency hypothyroidism  E03 Other hypothyroidism  E04 Other nontoxic goitre  E05 Thyrotoxicosis [hyperthyroidism]  E06 Thyroiditis  E07 Other disorders of thyroid |

| **Table S2.** Sample characteristics for the analytical sample and the remainder | | |  |
| --- | --- | --- | --- |
|  | **Baseline + FU questionnaire**  **(analytical sample, n=154,367)** | **Baseline only**  **(remainder, n=348,232)** |  |
|  | *Mean±SD or N(%)* | *Mean±SD or N(%)* | *p value* |
| Age *(median (IQR))* | 57 (50-62) | 58 (50-64) |  |
| Female | 87,210 (56.5) | 186,234 (53.5) | <0.001 |
| Deprivation* | -1.71±2.83 | -1.11±3.19 | <0.001 |
| Education level |  |  | <0.001 |
| *High* | 57,290 (37.2) | 85,307 (24.7) |  |
| *Intermediate* | 28,743 (18.7) | 76,445 (22.2) |  |
| *Low* | 67,880 (44.1) | 183,025 (53.1) |  |
| Employment status |  |  | <0.001 |
| *Employed* | 45,814 (29.8) | 121,342 (35.3) |  |
| *Retired* | 98,176 (64.0) | 189,101 (55.1) |  |
| *Unemployed*** | 9484 (6.2) | 33,171 (9.6) |  |
| Ethnicity |  |  | <0.001 |
| *White* | 149,449 (97.1) | 323,350 (93.5) |  |
| *ethnic minority* | 4918 (2.9) | 24,882 (6.5) |  |
| BMI *(kg/m^2^)* | 26.77±4.55 | 27.72±4.88 | <0.001 |
| Smoking status |  |  | <0.001 |
| *Past/current smoker* | 65,669 (42.5) | 161,052 (46.3) |  |
| *Never smoked* | 88,698 (57.5) | 187, 180 (53.7) |  |
| Alcohol intake *(units per week*) | 8.75±9.50 | 8.39±9.94 | <0.001 |
| Physical activity |  |  | <0.001 |
| *None* | 1732 (1.1) | 10,023 (2.9) |  |
| *Low* | 27,129 (17.6) | 72,085 (20.7) |  |
| *Moderate* | 64,704 (41.9) | 139,603 (40.1) |  |
| *Vigorous* | 60,802 (39.4) | 126,521 (36.3) |  |
| Baseline CRP level | 2.32±4.03 | 2.77±4.56 | <0.001 |
| Baseline antidepressant use | 8068 (5.2) | 23,403 (6.7) | <0.001 |
| Baseline depression |  |  | <0.001 |
| *Depression* | 18,974 (12.3) | 65,613 (18.8) |  |
| *No depression* | 135,393 (87.7) | 282,619 (81.2) |  |
| Baseline anxiety |  |  | <0.001 |
| *Anxiety* | 19,494 (12.6) | 70,050 (20.1) |  |
| *No anxiety* | 134, 873 (87.4) | 278,182 (79.9) |  |
| Physical Multimorbidity |  |  | <0.001 |
| *None or 1 LTC* | 110,529 (71.6) | 219,040 (62.9) |  |
| *2 LTCs* | 26,750 (17.3) | 69,022 (19.8) |  |
| *3 LTCs* | 10,996 (7.1) | 34,333 (9.9) |  |
| *4 LTCs* | 4003 (2.6) | 15,310 (4.4) |  |
| *≥ 5 LTCs* | 2089 (1.3) | 10,527 (3.0) |  |
| BMI=body mass index; GAD=General Anxiety Disorder; IQR=interquartile range; LTC=long-term conditions; PHQ=Patient Health Questionnaire  *Deprivation was measured using the Townsend Deprivation Index. Positive values indicate areas of high material deprivation, whereas negative values indicate relative affluence  **Unemployed includes carers, those in voluntary work, the long-term sick or disabled, and full-time students | | |  |

| **Table S3**. Rotated factor loadings for the 13 factors that emerged from exploratory factor analysis on physical long-term conditions | | | | | | | | | | | | | | |
| --- | --- | --- | --- | --- | --- | --- | --- | --- | --- | --- | --- | --- | --- | --- |
| **Variable** | **Factor 1** | **Factor 2** | **Factor 3** | **Factor 4** | **Factor 5** | **Factor 6** | **Factor 7** | **Factor 8** | **Factor 9** | **Factor 10** | **Factor 11** | **Factor 12** | **Factor 13** | **Uniqueness** |
| **Asthma** | 0.018 | 0.008 | -0.076 | -0.1668 | **0.6112** | **-0.4864** | 0.0173 | -0.0202 | -0.1759 | 0.0758 | -0.0237 | -0.0608 | 0.3659 | 0.3234 |
| **Atrial fibrillation** | -0.0615 | 0.1307 | 0.1628 | 0.014 | -0.0577 | 0.0862 | **0.6557** | 0.0839 | -0.182 | -0.0234 | -0.0311 | -0.0203 | -0.0115 | 0.4713 |
| **Bronchiectasis** | 0.1541 | -0.1187 | -0.0889 | -0.1171 | **0.7169** | 0.3084 | -0.1041 | -0.0572 | 0.0068 | -0.0808 | 0.041 | 0.182 | -0.0456 | 0.2479 |
| **Cancer** | -0.0068 | -0.0459 | -0.0569 | 0.0939 | 0.042 | -0.0835 | -0.0621 | 0.0657 | -0.0579 | 0.0411 | -0.132 | **0.8598** | -0.2594 | 0.3971 |
| **CFS** | **0.6005** | -0.0925 | 0.1373 | 0.0267 | 0.1054 | -0.031 | -0.2441 | -0.0731 | -0.0475 | 0.2008 | 0.1029 | -0.0408 | -0.0813 | 0.4537 |
| **CHD** | -0.0916 | **0.4516** | 0.0256 | -0.1483 | 0.0383 | 0.0808 | **0.481** | -0.0239 | -0.1571 | 0.1353 | 0.0025 | -0.1447 | -0.0081 | 0.4125 |
| **Chronic sinusitis** | -0.0662 | -0.0021 | 0.0254 | 0.1255 | 0.5826 | 0.0728 | 0.0506 | 0.1326 | 0.1131 | 0.1981 | 0.0434 | -0.1626 | -0.2018 | 0.458 |
| **CKD** | **0.4471** | 0.1882 | 0.1071 | -0.362 | -0.142 | 0.048 | 0.2523 | 0.0143 | -0.0086 | 0.1084 | -0.0603 | 0.2366 | -0.003 | 0.3478 |
| **Connective tissue disorders** | -0.0964 | -0.0725 | 0.0743 | -0.0146 | -0.0661 | 0.188 | 0.0634 | 0.0436 | 0.0988 | 0.0235 | 0.3623 | 0.0028 | **0.609** | 0.3658 |
| **COPD** | -0.0092 | 0.2198 | 0.0563 | -0.0883 | **0.7164** | -0.1756 | 0.0746 | 0.0013 | 0.0329 | -0.0103 | -0.0299 | 0.1147 | 0.0997 | 0.3835 |
| **Dementia** | -0.2168 | -0.2024 | -0.2934 | **-0.4556** | -0.2465 | -0.1138 | 0.105 | -0.0623 | -0.1143 | 0.0806 | 0.0922 | 0.0173 | -0.0805 | 0.0049 |
| **Diabetes** | -0.0462 | **0.8801** | -0.004 | -0.0478 | 0.105 | 0.0697 | 0.0491 | 0.0125 | 0.2115 | -0.115 | 0.1537 | -0.1464 | -0.0292 | 0.3117 |
| **Diverticular disease** | -0.047 | 0.2539 | 0.0572 | 0.3531 | -0.0887 | 0.0488 | -0.1266 | -0.0513 | -0.1412 | 0.3612 | -0.0053 | 0.2144 | 0.0777 | 0.5022 |
| **Dyspepsia** | -0.0802 | 0.0566 | 0.105 | -0.0497 | 0.09 | -0.02 | 0.1612 | -0.0983 | 0.0226 | **0.5999** | 0.0929 | 0.0387 | -0.0415 | 0.5418 |
| **Endometriosis** | -0.1443 | 0.0486 | 0.0214 | 0.1291 | 0.0748 | 0.083 | 0.0135 | **0.9615** | -0.0578 | -0.0002 | -0.0702 | 0.0799 | 0.0198 | 0.0991 |
| **Epilepsy** | **0.762** | -0.0911 | -0.0251 | 0.2159 | -0.0063 | -0.1171 | 0.3092 | -0.1524 | 0.1402 | -0.1531 | -0.1371 | 0.0369 | 0.0223 | 0.3227 |
| **Glaucoma** | -0.0132 | 0.3365 | -0.1417 | **0.6225** | -0.0243 | -0.0776 | -0.0656 | -0.0694 | -0.1335 | -0.2298 | 0.1788 | 0.2638 | 0.0343 | 0.4891 |
| **Heart failure** | 0.2137 | 0.0819 | 0.114 | 0.0728 | 0.1263 | 0.2885 | 0.2546 | 0.0665 | **-0.7052** | -0.1115 | -0.0228 | -0.0335 | -0.0502 | 0.069 |
| **Hepatitis** | -0.0293 | -0.2358 | **0.9575** | -0.0325 | -0.0269 | -0.1469 | -0.014 | -0.0708 | -0.1129 | -0.0647 | 0.0567 | -0.1636 | -0.0663 | 0.2734 |
| **Hypertension** | -0.0196 | **0.7125** | -0.2556 | -0.0108 | -0.0242 | 0.0056 | 0.3137 | -0.0217 | 0.0929 | 0.1152 | 0.0578 | -0.0049 | -0.0355 | 0.4283 |
| **IBD** | -0.0535 | 0.1667 | 0.281 | -0.0446 | 0.047 | 0.0512 | -0.1361 | 0.0806 | 0.0488 | 0.2616 | -0.0937 | 0.2827 | 0.0969 | 0.5567 |
| **IBS** | 0.0654 | -0.1092 | -0.1096 | -0.013 | -0.0304 | 0.0395 | -0.1159 | 0.0223 | 0.0295 | **0.7731** | -0.1198 | 0.016 | 0.0408 | 0.3838 |
| **Liver disease** | -0.0039 | 0.0007 | **0.8042** | -0.0266 | -0.0152 | -0.0144 | 0.2032 | -0.0167 | 0.0814 | -0.0253 | 0.0704 | 0.1214 | 0.06 | 0.2948 |
| **Meniere’s disease** | -0.0475 | -0.0926 | 0.0006 | **0.7747** | -0.1622 | 0.0289 | 0.1482 | -0.0268 | -0.0985 | 0.0718 | 0.0986 | 0.0564 | 0.1065 | 0.5409 |
| **Migraine** | 0.1386 | -0.2694 | -0.0226 | **0.535** | 0.0466 | 0.0951 | 0.156 | 0.1224 | 0.0958 | 0.232 | 0.0235 | -0.1093 | 0.0224 | 0.5034 |
| **MS** | 0.0192 | 0.2606 | 0.0221 | -0.0944 | 0.091 | 0.1091 | -0.0202 | -0.0299 | **0.9816** | -0.052 | -0.0259 | -0.0363 | -0.0326 | 0.1329 |
| **Osteoporosis** | 0.015 | -0.3452 | 0.0262 | 0.0938 | 0.1385 | 0.0606 | 0.1011 | 0.0213 | 0.1256 | 0.0071 | 0.2695 | **0.5452** | 0.1521 | 0.3514 |
| **Painful conditions** | 0.0176 | 0.0325 | -0.1148 | 0.2142 | 0.0676 | -0.1496 | 0.2767 | -0.0625 | -0.0672 | **0.5148** | 0.1724 | -0.0097 | -0.0676 | 0.5915 |
| **Parkinson’s disease** | **-0.9322** | 0.0206 | 0.1241 | 0.1125 | -0.0429 | -0.1512 | 0.0882 | -0.0346 | 0.143 | 0.0394 | -0.1311 | 0.0148 | 0.0608 | 0.0262 |
| **PCOS** | 0.3943 | 0.1008 | 0.094 | 0.0648 | -0.1732 | **-0.6139** | -0.0679 | 0.3483 | 0.1503 | 0.048 | 0.0398 | -0.0437 | 0.0023 | 0.1079 |
| **Pernicious anaemia** | 0.3309 | 0.0973 | -0.0482 | -0.0508 | -0.0516 | 0.0805 | -0.1709 | -0.0167 | -0.0696 | 0.0963 | **0.699** | -0.1434 | 0.0933 | 0.3277 |
| **Prostate conditions** | 0.0217 | 0.1151 | 0.1311 | 0.1813 | 0.0178 | 0.1446 | -0.0678 | **-0.8974** | 0.0388 | 0.1152 | -0.1135 | -0.0266 | -0.0096 | 0.0659 |
| **Psoriasis/eczema** | -0.0358 | -0.0076 | -0.0382 | 0.2678 | 0.0289 | 0.077 | -0.0889 | 0.0042 | -0.0535 | -0.0412 | -0.1613 | -0.2589 | **0.8034** | 0.3122 |
| **PVD** | 0.1353 | 0.1265 | -0.0927 | 0.0675 | -0.0761 | **0.8242** | 0.0563 | 0.031 | 0.043 | 0.0161 | -0.0466 | -0.0953 | 0.1653 | 0.2637 |
| **Stroke/TIA** | 0.0605 | 0.0212 | -0.0241 | 0.202 | 0.0588 | -0.0053 | **0.8282** | 0.0035 | 0.0427 | 0.048 | -0.1132 | 0.0029 | -0.0247 | 0.3742 |
| **Thyroid problems** | -0.0627 | 0.2041 | 0.1061 | 0.2269 | 0.0446 | -0.1427 | -0.0466 | 0.0236 | 0.0034 | -0.0358 | **0.8644** | -0.0089 | -0.0973 | 0.3638 |

| **Table S4.** Cross-sectional associations between physical multimorbidity and common mental health disorders at baseline (n=152,869) | | | | | | |
| --- | --- | --- | --- | --- | --- | --- |
| Depression | | | | | | |
|  | Unadjusted | | Age and sex adjusted | | Fully adjusted* | |
|  | *OR (95% CI)* | *p value* | *OR (95% CI)* | *p value* | *OR (95% CI)* | *p value* |
| Physical multimorbidity |  |  |  |  |  |  |
| *No multimorbidity* | Reference |  | Reference |  | Reference |  |
| *Two conditions* | 1.29 (1.24 to 1.34) | <0.001 | 1.42 (1.37 to 1.48) | <0.001 | 1.09 (1.05 to 1.15) | <0.001 |
| *Three conditions* | 1.61 (1.53 to 1.70) | <0.001 | 1.86 (1.76 to 1.97) | <0.001 | 1.17 (1.09 to 1.24) | <0.001 |
| *Four conditions* | 1.94 (1.79 to 2.10) | <0.001 | 2.28 (2.10 to 2.47) | <0.001 | 1.14 (1.03 to 1.26) | 0.008 |
| *≥ Five conditions* | 2.68 (2.43 to 2.97) | <0.001 | 3.21 (2.90 to 3.56) | <0.001 | 1.15 (1.01 to 1.31) | 0.029 |
| Anxiety | | | | | | |
|  | Unadjusted | | Age and sex adjusted | | Fully adjusted* | |
|  | *OR (95% CI)* | *p value* | *OR (95% CI)* | *p value* | *OR (95% CI)* | *p value* |
| Physical multimorbidity |  |  |  |  |  |  |
| *No multimorbidity* | Reference |  | Reference |  | Reference |  |
| *Two conditions* | 1.44 (1.38 to 1.50) | <0.001 | 1.60 (1.54 to 1.66) | <0.001 | 1.40 (1.34 to 1.46) | <0.001 |
| *Three conditions* | 1.90 (1.81 to 2.00) | <0.001 | 2.21 (2.10 to 2.33) | <0.001 | 1.74 (1.64 to 1.84) | <0.001 |
| *Four conditions* | 2.43 (2.25 to 2.62) | <0.001 | 2.89 (2.67 to 3.12) | <0.001 | 2.05 (1.88 to 2.24) | <0.001 |
| *≥ Five conditions* | 3.63 (3.30 to 3.99) | <0.001 | 4.39 (3.99 to 4.84) | <0.001 | 2.64 (2.37 to 2.95) | <0.001 |
| *Covariates: Age, sex, deprivation, education level, employment status, ethnicity, BMI, smoking status, alcohol intake, physical activity, CRP levels, antidepressant use, baseline anxiety (for depression analysis), baseline depression (for anxiety analysis) | | | | | | |

| **Table S5.** Sample characteristics for each physical multimorbidity cluster | | | | | | | | |  |  |
| --- | --- | --- | --- | --- | --- | --- | --- | --- | --- | --- |
|  | **No multimorbidity**  **(n=110,529)** | **Undefined multimorbidity**  **(n=31,173)** | **Cardiometabolic**  **(n=5101)** | **Respiratory**  **(n=727)** | **Cardio/**  **Cerebrovascular**  **(n=621)** | **Reproductive**  **(n=3055)** | **Pain/**  **Gastrointestinal**  **(n=4999)** | | |  |
|  | *Mean±SD or N(%)* | *Mean±SD or N(%)* | *Mean±SD or N(%)* | *Mean±SD or N(%)* | *Mean±SD or N(%)* | *Mean±SD or N(%)* | *Mean±SD or N(%)* | | |  |
| Age *(median (IQR))* | 55 (49-61) | 60 (53-64) | 62 (57-65) | 60 (55-64) | 63 (59-67) | 62 (56-66) | 60 (54-64) | | |  |
| Female | 62,471 (56.5) | 18,643 (59.8) | 1567 (30.7) | 403 (55.4) | 133 (21.4) | 852 (27.9) | 3205 (64.1) | | |  |
| Deprivation* | -1.74±2.82 | -1.70±2.81 | -1.30±3.04 | -1.05±3.15 | -1.17±3.14 | -1.81±2.81 | -1.57±2.92 | | |  |
| Education level |  |  |  |  |  |  |  | | |  |
| *High* | 40,907 (37.0) | 11,868 (38.1) | 1677 (32.9) | 243 (33.4) | 206 (33.2) | 1154 (37.8) | 1868 (37.4) | | |  |
| *Intermediate* | 21,472 (19.4) | 5612 (18.0) | 2433 (47.7) | 159 (21.9) | 105 (16.9) | 572 (18.7) | 968 (19.4) | | |  |
| *Low* | 48,150 (43.6) | 13,683 (43.9) | 991 (19.4) | 325 (44.7) | 310 (49.9) | 1329 (43.5) | 2163 (43.2) | | |  |
| Employment status |  |  |  |  |  |  |  | | |  |
| *Employed* | 28,037 (25.4) | 12,178 (39.1) | 2509 (49.2) | 308 (42.4) | 358 (57.6) | 1453 (47.6) | 2135 (42.7) | | |  |
| *Retired* | 76,260 (69.0) | 16,887 (54.2) | 2164 (42.4) | 329 (45.2) | 189 (30.4) | 1412 (46.2) | 2318 (46.4) | | |  |
| *Unemployed*** | 6232 (5.6) | 2108 (6.7) | 428 (8.4) | 90 (12.4) | 74 (11.9) | 190 (6.2) | 546 (10.9) | | |  |
| Ethnicity |  |  |  |  |  |  |  | | |  |
| *White* | 107,192 (97.0) | 30,450 (97.7) | 4875 (95.6) | 714 (98.2) | 611 (98.4) | 2981 (97.6) | 4883 (97.7) | | |  |
| *Ethnic minority* | 3337 (3.0) | 723 (2.3) | 226 (4.4) | 13 (1.8) | 10 (1.6) | 74 (2.4) | 116 (2.3) | | |  |
| BMI *(kg/m^2^)* | 26.23±4.17 | 27.72±4.93 | 30.92±5.71 | 28.1±5.65 | 29.43±5.03 | 27.55±4.39 | 28.64±5.30 | | |  |
| Smoking status |  |  |  |  |  |  |  | | |  |
| *Past/current smoker* | 44,667 (40.4) | 14,388 (46.2) | 2936 (57.6) | 437 (60.1) | 389 (62.6) | 1503 (49.2) | 2411 (48.2) | | |  |
| *Never smoked* | 65,862 (59.6) | 16,785 (53.8) | 2165 (42.4) | 290 (39.9) | 232 (37.4) | 1552 (50.8) | 2588 (51.8) | | |  |
| Alcohol intake *(units per week*) | 20.82±16.70 | 21.20±17.20 | 25.96±19.85 | 23.93±20.08 | 26.54±17.87 | 22.84±16.06 | 21.31±17.30 | | |  |
| Physical activity |  |  |  |  |  |  |  | | |  |
| *None* | 928 (0.8) | 477 (1.5) | 159 (3.1) | 21 (2.9) | 17 (2.7) | 37 (1.21) | 151 (3.0) | | |  |
| *Low* | 18,379 (16.6) | 6027 (19.3) | 1188 (23.3) | 179 (24.6) | 131 (21.1) | 573 (18.8) | 1034 (20.7) | | |  |
| *Moderate* | 46,129 (41.7) | 13,245 (42.5) | 2152 (42.2) | 279 (38.4) | 267 (43.0) | 1300 (42.5) | 2082 (41.7) | | |  |
| *Vigorous* | 45,093 (40.8) | 11,424 (36.6) | 1602 (31.4) | 248 (34.1) | 206 (33.2) | 1145 (37.5) | 1732 (34.6) | | |  |
| Baseline CRP level | 2.06±3.52 | 2.85±4.62 | 3.16±4.70 | 4.32±6.90 | 3.18±4.79 | 2.69±4.66 | 3.36±4.89 | | |  |
| Baseline antidepressant use | 4291 (3.9) | 2334 (7.5) | 462 (9.1) | 85 (11.7) | 48 (7.7) | 242 (7.9) | 800 (16.0) | | |  |
| Baseline depression |  |  |  |  |  |  |  | | |  |
| *Depression* | 12,169 (11.0) | 4552 (14.6) | 742 (14.5) | 153 (21.1) | 80 (12.9) | 462 (15.1) | 1118 (22.4) | | |  |
| *No depression* | 98,360 (89.0) | 26,621 (85.4) | 4359 (85.5) | 574 (78.9) | 541 (87.1) | 2593 (84.9) | 3881 (77.6) | | |  |
| PHQ-9 follow-up | 2.51±3.43 | 3.21±4.04 | 3.64±4.55 | 4.62±5.03 | 3.72±4.50 | 3.16±4.11 | 4.42±4.90 | | |  |
| PHQ-9 depression |  |  |  |  |  |  |  | | |  |
| *Depression* | 5175 (4.7) | 2313 (7.4) | 523 (10.2) | 113 (15.5) | 63 (10.1) | 222 (7.3) | 668 (13.4) | | |  |
| *No depression* | 105,354 (95.3) | 28,860 (92.6) | 4578 (89.8) | 614 (84.5) | 558 (89.9) | 2833 (92.7) | 4331 (86.6) | | |  |
| Baseline anxiety |  |  |  |  |  |  |  | | |  |
| *Anxiety* | 11,924 (10.8) | 4969 (15.9) | 903 (17.7) | 190 (26.1) | 126 (20.3) | 471 (15.4) | 1236 (24.7) | | |  |
| *No anxiety* | 98,605 (89.2) | 26,204 (84.1) | 4198 (82.3) | 537 (73.9) | 495 (79.7) | 2584 (84.6) | 3763 (75.3) | | |  |
| GAD-7 follow-up | 1.98±3.18 | 2.37±3.62 | 2.30±3.75 | 3.06±4.25 | 2.15±3.48 | 2.19±3.59 | 3.22±4.28 | | |  |
| GAD-7 anxiety |  |  |  |  |  |  |  | | |  |
| *Anxiety* | 4037 (3.6) | 1620 (5.2) | 285 (5.6) | 62 (8.5) | 30 (4.8) | 144 (4.7) | 426 (8.5) | | |  |
| *No anxiety* | 106,492 (96.4) | 29,553 (94.8) | 4816 (94.4) | 665 (91.5) | 591 (95.2) | 2911 (95.3) | 4573 (91.5) | | |  |
| Physical Multimorbidity |  |  |  |  |  |  |  | | |  |
| *None or 1 LTC* | 110,529 | - | - | - | - |  | - | | |  |
| *2 LTCs* | - | 23,288 (74.7) | 1627 (31.9) | 158 (21.7) | 64 (10.3) | 1585 (51.9) | 1365 (27.3) | | |  |
| *3 LTCs* | - | 6161 (19.8) | 1691 (33.1) | 227 (31.2) | 154 (24.8) | 654 (21.4) | 1689 (33.8) | | |  |
| *4 LTCs* | - | 1425 (4.6) | 972 (19.1) | 151 (20.8) | 183 (29.5) | 492 (16.1) | 1054 (21.1) | | |  |
| *≥ 5 LTCs* | - | 299 (0.9) | 811 (15.9) | 191 (26.3) | 220 (35.4) | 324 (10.6) | 891 (17.8) | | |  |
| Follow-up duration *(years)* | 7.61±0.88 | 7.58±0.86 | 7.56±0.86 | 7.56±0.84 | 7.56±0.83 | 7.55±0.86 | 7.57±0.83 | | |  |
| BMI=body mass index; GAD=General Anxiety Disorder; IQR=interquartile range; LTC=long-term conditions; PHQ=Patient Health Questionnaire  *Deprivation was measured using the Townsend Deprivation Index. Positive values indicate areas of high material deprivation, whereas negative values indicate relative affluence  **Unemployed includes carers, those in voluntary work, the long-term sick or disabled, and full-time students  Note: Although 12,665 participants belonged to multimorbidity patterns, the sample size for each multimorbidity pattern amount to 15,444 reflecting a certain amount of overlap between patterns, i.e., participants can belong to one or more patterns | | | | | | | |  |  |  |

| **Table S6.** Cross-sectional associations between physical multimorbidity patterns and common mental health disorders at baseline (n=154,367) | | | | | | | |
| --- | --- | --- | --- | --- | --- | --- | --- |
| Depression | | | | | | | |
|  | Unadjusted | | Age and sex adjusted | | | Fully adjusted* | |
|  | *OR (95% CI)* | *p value* | *OR (95% CI)* | | *p value* | *OR (95% CI)* | *p value* |
| Physical multimorbidity |  |  |  | |  |  |  |
| *No multimorbidity* | Reference |  | Reference | |  | Reference |  |
| *Undefined multimorbidity* | 1.38 (1.33 to 1.43) | <0.001 | 1.51 (1.45 to 1.56) | | <0.001 | 1.23 (1.18 to 1.28) | <0.001 |
| *Cardiometabolic* | 1.37 (1.27 to 1.49) | <0.001 | 1.75 (1.61 to 1.90) | | <0.001 | 1.16 (1.06 to 1.28) | 0.001 |
| *Respiratory* | 2.15 (1.80 to 2.58) | <0.001 | 2.42 (2.02 to 2.90) | | <0.001 | 1.46 (1.20 to 1.79) | <0.001 |
| *Cardio/cerebrovascular* | 1.19 (0.94 to 1.51) | 0.138 | 1.62 (1.28 to 2.06) | | <0.001 | 0.96 (0.74 to 1.25) | 0.790 |
| *Reproductive* | 1.44 (1.30 to 1.59) | <0.001 | 1.79 (1.62 to 1.99) | | <0.001 | 1.47 (1.32 to 1.65) | <0.001 |
| *Pain/gastrointestinal* | 2.33 (2.17 to 2.49) | <0.001 | 2.54 (2.37 to 2.73) | | <0.001 | 1.68 (1.55 to 1.82) | <0.001 |
| Anxiety |  |  |  | |  |  |  |
|  | Unadjusted | | Age and sex adjusted | | | Fully adjusted* | |
|  | *OR (95% CI)* | *p value* | *OR (95% CI)* | *p value* | | *OR (95% CI)* | *p value* |
| Physical multimorbidity |  |  |  |  | |  |  |
| *No multimorbidity* | Reference |  | Reference |  | | Reference |  |
| *Undefined multimorbidity* | 1.56 (1.51 to 1.62) | <0.001 | 1.71 (1.65 to 1.78) | <0.001 | | 1.49 (1.43 to 1.55) | <0.001 |
| *Cardiometabolic* | 1.78 (1.65 to 1.92) | <0.001 | 2.26 (2.09 to 2.44) | <0.001 | | 1.73 (1.59 to 1.89) | <0.001 |
| *Respiratory* | 2.92 (1.48 to 3.46) | <0.001 | 3.29 (2.78 to 3.89) | <0.001 | | 2.46 (2.04 to 2.97) | <0.001 |
| *Cardio/cerebrovascular* | 2.10 (1.73 to 2.56) | <0.001 | 2.87 (2.35 to 3.50) | <0.001 | | 2.44 (1.96 to 3.03) | <0.001 |
| *Reproductive* | 1.51 (1.36 to 1.66) | <0.001 | 1.88 (1.70 to 2.08) | <0.001 | | 1.58 (1.41 to 1.76) | <0.001 |
| *Pain/gastrointestinal* | 2.72 (2.54 to 2.90) | <0.001 | 2.97 (2.77 to 3.18) | <0.001 | | 2.18 (2.02 to 2.35) | <0.001 |
| * Covariates: Age, sex, deprivation, education level, employment status, ethnicity, BMI, smoking status, alcohol intake, physical activity, CRP level, baseline anxiety (for depression analysis), baseline depression (for anxiety analysis) | | | | | | | |
